# Supplementary material for: Development of High-Throughput Multiplex Serology to Detect Serum Antibodies against Coxiella burnetii
Source: Microorganisms. 2021 Nov 17;9(11):2373. doi: 10.3390/microorganisms9112373 (PMC8623512; doi:10.3390/microorganisms9112373)
Supplement: Supplementary file 1 [file microorganisms-09-02373-s001.zip › microorganisms-1439063-supplementary.pdf]

Supplementary Table S1: Quantitative seroresponses to nine selected *C. burnetii* antigens measured with Multiplex serology in a serum dilution of 1:1000. Patients were grouped according to a positive (pos.) or negative (neg.) *C. burnetii* reference assay test result. Groups were compared using Mann-Whitney U test. A p-value below 0.05 is considered statistically significant.

Antigen locus tag	Antigen symbol	*C. burnetii* pos. [MFI] (SD)	*C. burnetii* neg. [MFI] (SD)	p-value
CBU_0092	YbgF	7 (22)	10 (41)	0.99[1]
CBU_0937		7 (6)	15 (17)	<0.01[1]
CBU_0370		6 (6)	12 (11)	<0.01[1]
CBU_0952	AdaA	21 (99)	22 (106)	0.1[1]
**CBU_1910**	**Com1**	**173 (267)**	**16 (67)**	**<0.001**
**CBU_1718**	**GroEL**	**172 (229)**	**160 (612)**	**<0.001**
**CBU_1290**	**DnaK**	**14 (31)**	**38 (225)**	**<0.05**
CBU_0630	Mip	22 (101)	18 (109)	0.79[1]
CBU_1425		46 (88)	71 (217)	0.20

[1] Most MFI values fall below the lower limit of quantitation of 30 MFI

Supplementary Table S2: Performance of nine *C. burnetii* antigens measured in 76 patients with positive and 91 patients with negative *C. burnetii* serostatus in a serum dilution of 1:1000. Numerical measurement values were dichotomized using the given cutoff (maximum value of the Youden's index) to calculate the respective assay characteristics.

Antigen locus tag	Antigen symbol	Cutoff [MFI]	Specificity [%]	Sensitivity [%]	Cohen's Kappa κ (95% CI)
CBU_0092	YbgF	30[1]	97	7	0.04 (-0.13 to 0.20)
CBU_0937		30[1]	92	0	-0.08 (-0.25 to 0.08)
CBU_0370		30[1]	91	0	-0.09 (-0.26 to 0.07)
CBU_0952	AdaA	30[1]	93	7	0.00 (-0.16 to 0.16)
**CBU_1910**	**Com1**	**43**	**97**	**59**	**0.58 (0.45 to 0.70)**
**CBU_1718**	**GroEL**	**39**	**73**	**75**	**0.47 (0.43 to 0.61)**
**CBU_1290**	**DnaK**	**30[1]**	**95**	**13**	**0.21 (0.06 to 0.36)**
CBU_0630	Mip	33	97	8	0.05 (-0.11 to 0.21)
CBU_1425		41	36	80	-0.16 (-0.31 to 0.00)

[1] A technical cutoff of 30 MFI was applied to pass the lower limit of quantitation

Supplementary Table S3: Performance of nine *C. burnetii* antigens measured in 28 patients with a positive *C. burnetii* serostatus and a phase I endpoint titer ≥1:1,024 and 91 patients with a negative *C. burnetii* serostatus in a serum dilution of 1:1000. Numerical measurement values were dichotomized using the given cutoff (maximum value of the Youden's index) to calculate the respective assay characteristics.

Antigen		Cutoff [MFI]	Specificity [%]	Sensitivity [%]	Cohen's Kappa κ (95% CI)
CBU_0092	YbgF	30[1]	97	11	0.10 (-0.19 to 0.39)
CBU_0937		30[1]	92	0	-0.10 (-0.41 to 0.20)
CBU_0370		30[1]	91	0	-0.12 (-0.42 to 0.19)
CBU_0952	AdaA	30[1]	93	0	-0.09 (-0.40 to 0.22)
**CBU_1910**	**Com1**	**30[1]**	**95**	**79**	**0.74 (0.59 to 0.89)**
**CBU_1718**	**GroEL**	**40**	**73**	**93**	**0.51 (0.35 to 0.67)**
CBU_1290	DnaK	30[1]	95	25	0.24 (-0.01 to 0.50)
CBU_0630	Mip	30[1]	96	4	-0.01 (-0.32 to 0.29)
CBU_1425		30[1]	53	32	-0.12 (-0.31 to 0.08)

[1] A technical cutoff of 30 MFI was applied to pass the lower limit of quantitation

Supplementary Table S4: Multiplex Serology assay characteristics and performance of antigens Com1 and GroEL, as well as a combination of both antigens in a serum dilution of 1:1000. Assay performances were determined in an overall *C. burnetii* positive population (acute and chronic Q fever) and a subgroup of them who show a high phase I endpoint titer of ≥1:1,024, as an approximation for chronic Q fever.

Seropositive reference population	Antigen	Cutoff [MFI]	Specificity [%]	Sensitivity [%]
76 *C. burnetii* seropositive patients	Com1 (CBU_1910)	30[1]	95	79
	GroEL (CBU_1718)	150	90	41
	Com 1 + GroEL (double positives)	30[1]/30[1]	98	57
28 *C. burnetii* seropositive patients with a Phase I titer ≥1:1,024	Com1 (CBU_1910)	30[1]	95	79
	GroEL (CBU_1718)	150	90	54
	Com 1 + GroEL (double positives)	30[1]/30[1]	98	79

[1] A technical cutoff of 30 MFI was applied to pass the lower limit of quantitation

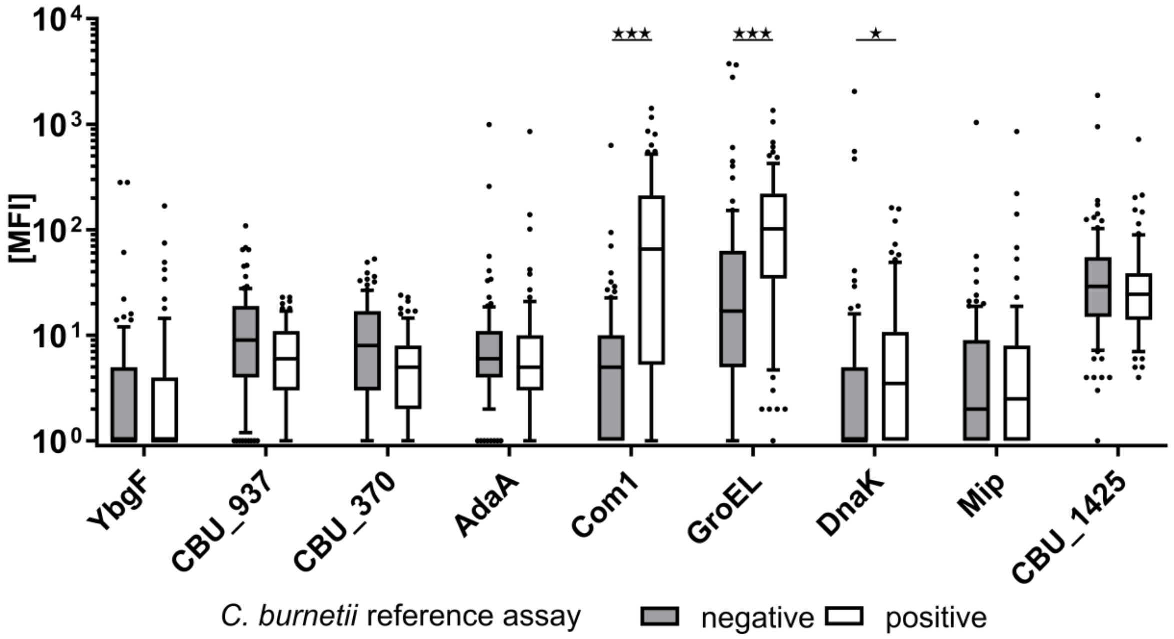

Supplementary Figure S1: Antibody levels in the reference panel of 76 *C. burnetii* seropositive and 91 seronegative patients measured in a serum dilution of 1:1000. Com1 shows a significantly different distribution ($p < 0.05$) between the two reference groups.

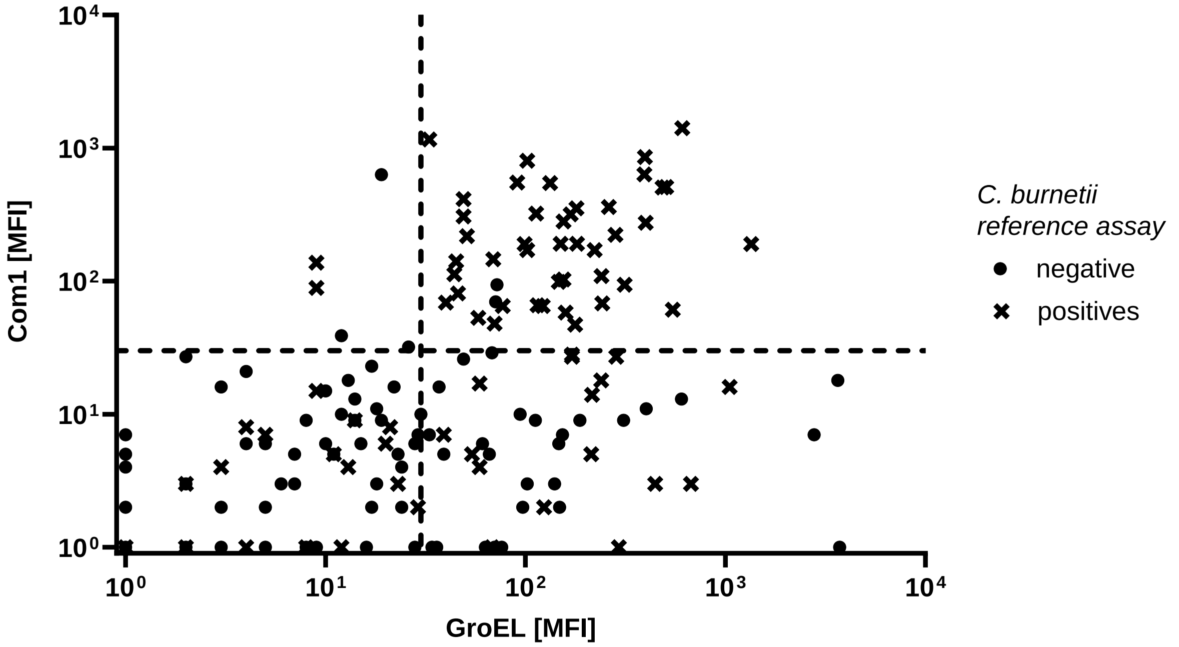

Supplementary Figure S2: Antibody responses against the *C. burnetii* antigens Com1 and GroEL measured in 76 *C. burnetii* seropositive and 91 seronegative patients in a serum dilution of 1:1000. Dashed lines (x = 30 MFI; y = 30 MFI) indicate the respective cutoffs to obtain a specificity of 98% and combined sensitivity of 57%.
